# *In situ* microwave heating fabrication of copper nanoparticles inside cotton fiber using pressurization in immiscible liquids with raw material solutions†

**DOI:** 10.1039/d1ra04868f

**Published:** 2021-10-04

**Authors:** Masato Miyakawa, Chizuru Shigaraki, Takashi Nakamura, Masateru Nishioka

**Affiliations:** National Institute of Advanced Industrial Science and Technology, AIST 4-2-1, Nigatake, Miyagino-ku Sendai 983-8551 Japan m-nishioka@aist.go.jp

## Abstract

We developed a method for *in situ* fabrication of copper nanoparticles inside cotton fibers. Copper nanoparticles can be fabricated mainly in the central part of the fiber by absorbing a raw material solution and by applying microwave heating in a state where the raw material solution is pressed with immiscible liquids. Surface SEM images and cross-sectional EDS mapping for the fabricated fibers clarified that copper nanoparticles fabricated on the cotton surface were suppressed considerably more by the hydrophobic raw material solution than by the hydrophilic raw material solution. These cotton fibers containing copper nanoparticles were found to have antiviral properties against the influenza A virus.

## Abstract 2

Copper nanoparticles were created inside of cotton fibers by pressuring immiscible liquids against raw material solutions and applying microwave heating.

## Graphical abstract

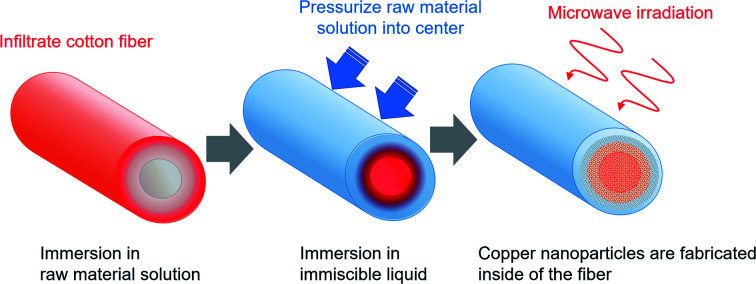

## Introduction

Cotton, the most widely used of natural fibers, is applied for various products such as clothing and housing to add functionality such as antibacterial action and water repellency.^1–6^ Recently, environmental loads imposed by plastic products have created difficulties. Worldwide interest in a shift to natural fibers from petroleum-derived synthetic fibers has increased.^7–9^ Methods for imparting functionality to natural fibers have mainly included impregnation and coating methods.^10,11^ These methods mainly apply functionality to the fiber surface. Therefore, changes in the fiber texture and performance deterioration have occurred because of friction and wear. In addition, natural fibers have many opportunities to contact human skin. Therefore, high demand has arisen for a method of imparting functionality inside of the fibers. As one example of a functional material inside a cotton fiber, a method of creating silver nanoparticles within microfibrillar substructures has been reported.^12,13^ Nevertheless, no versatile method to install diverse functional materials inside of natural fibers has been reported.

**Fig. 1:**
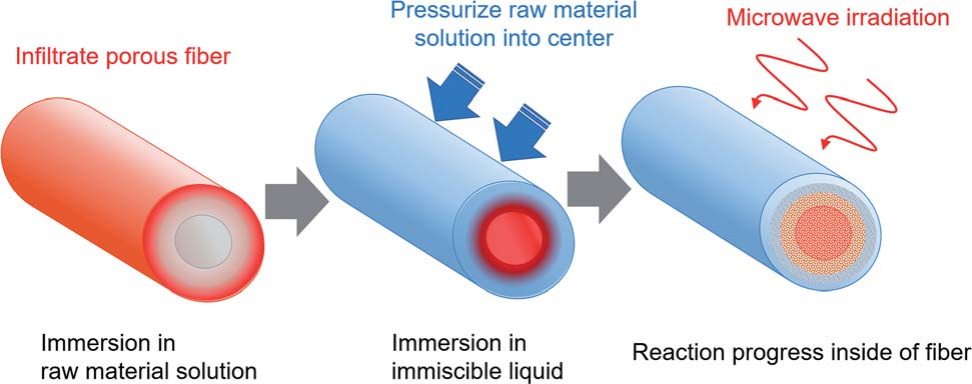
Schematic drawing of the fabrication method for functional materials inside porous fibers.

After developing microwave (MW) heating equipment, we considered the use of MW heating characteristics in a wider range of fields.^14–18^ We specifically examined characteristics of selective heating using MWs. Such characteristics depend on the dielectric constant of the substance. Moreover, we devised a method for selectively installing a functional material inside of porous fibers (Fig. 1).^19^ For this method, the porous fiber is first immersed in the raw material solution, which permeates the entire fiber. After the fiber is removed, it is immersed in a liquid that is immiscible with the raw material solution and which is pressurized to transfer the raw material solution into the fiber. Finally, while maintaining the pressurized state, a chemical reaction occurs in the raw material solution during MW or other heating. Results suggest that selective heating can be achieved using a substance in the raw material solution that is heated easily by MWs. The functional material can be installed inside the porous fibers. Reaction operations are not limited to MW heating. They might include holding of the raw materials at room temperature. As a model reaction, we fabricated silver-nanoparticle-containing cotton fibers or fibers with zeolite particles inside of porous polytetrafluoroethylene (PTFE) fibers.

Nanomaterials have been studied in various fields, especially in the field of nanomedicines, in diverse applications related to disease diagnosis and treatment such as drug delivery, biosensors, and cancer therapeutic agents.^20–24^ Copper and copper-oxide nanoparticles, which are typical nanomaterials, have numerous useful properties: they are UV protection and photocatalytic, particularly providing antibacterial and conductivity. Numerous methods for applying them to cotton fibers have been reported.^25–39^ In addition, because of the antiviral properties of copper and copper-oxide nanoparticles, interest in them is growing as a potential countermeasure against the COVID-19 pandemic.^40–42^ Nanomaterials and nanomaterial complexes of the future will be applied in various situations of daily life such as personal protective equipment and antiviral products in public places, in addition to medical use as nanomedicines such as drug and vaccine delivery.^43–49^

For this study, we performed selective installation of copper nanoparticles inside cotton fibers using MW heating in a state of pressurization with immiscible liquids in the raw material solution. No report of the relevant literature has described an example of the selective introduction of copper nanoparticles inside cotton fibers. The fabricated cotton fiber characteristics were compared using raw material solutions of two types: a hydrophilic ethylene glycol (EG) solution and a hydrophobic octanol solution. Structural analyses of copper nanoparticles and cotton fibers were performed using X-ray diffraction (XRD), transmission electron microscopy (TEM), and infrared absorption spectrometry (ATR-FTIR). The distribution of copper components in the fibers was ascertained using scanning electron microscopy-energy dispersive X-ray spectrometry (SEM-EDS). Antiviral tests for fabricated fibers were also conducted using influenza A virus.

## Experimental

### Fabrication of copper nanoparticles inside cotton fibers

Table 1 presents a list of experiment conditions. For the creation of copper nanoparticles using a hydrophilic raw material solution, a cotton cloth (35 mm × 600 mm, 2.3 g) was immersed in EG solution Cu(CH_3_COO)_2_ (10 or 100 mM) for 1 min. As the cotton cloth, a standard cloth for antibacterial evaluation was obtained for use from the Japan Textile Evaluation Technology Council. Then this cotton cloth was compressed lightly to make its weight approximately twice that of the dried cotton cloth. Next, this cotton cloth, dodecane, and hexane were placed in a PTFE vessel (100 ml capacity). The cotton cloth was immersed in the mixed solvent to conduct MW heating (MicroSYNTH; Milestone Inc). Typical heating conditions were 220 °C for 5 min. The vessel pressure was *ca.* 1.5 MPa at 220 °C. The vessel pressure was controllable by changing the mixing ratios of dodecane (bp 215 °C) and hexane (bp 68 °C). Under conditions of a large ratio of hexane, the inner pressure was higher. After cooling the vessel to 50 °C, the cotton cloth was removed and then washed for 10 min in ethanol using an ultrasonic cleaner. Then it was dried naturally in air at room temperature for 24 h. Synthesis was conducted identically using Cu(NO_3_)_2_·3H_2_O (100 mM) as a copper raw material. As a reference condition, the hydrophilic copper raw material solution was absorbed similarly using a cotton cloth. It was then heated at 220 °C for 30 min in an electric furnace.

**Table 1:** Fabrication conditions for cotton fibers containing copper nanoparticles

Entry no.	Raw material solution	Fiber weight ratio^a^	Immiscible liquids	Reaction operation	Reaction conditions	Maximum internal pressure (MPa)
1	EG, Cu(CH_3_COO)_2_, 100 mM	2.4–2.5	Dodecane (30 ml) and hexane (10 ml)	MW heating	220 °C, 5 min	1.5
2 (ref.)	EG, Cu(CH_3_COO)_2_, 100 mM	2.4–2.5	—	Electric furnace	220 °C, 30 min	—
3	EG, Cu(CH_3_COO)_2_, 10 mM	1.7–1.8	Dodecane (30 ml) and hexane (10 ml)	MW heating	220 °C, 5 min	1.4
4	EG, Cu(NO_3_)_2_·3H_2_O, 100 mM	2.4–2.5	Dodecane (30 ml) and hexane (10 ml)	MW heating	220 °C, 5 min	1.7
5	1-Octanol, Cu(C_2_H_5_COO)_2_·H_2_O, 100 mM	1.7–1.8	EG (40 ml) and hexane (3 ml)	MW heating	220 °C, 5 min	1.4
6	1-Octanol, Cu(C_5_H_11_COO)_2_, 100 mM	1.7–1.8	EG (40 ml) and hexane (3 ml)	MW heating	220 °C, 5 min	1.4
7	1-Octanol, Cu(C_5_H_11_COO)_2_, 30 mM	1.7–1.8	EG (40 ml) and hexane (3 ml)	MW heating	220 °C, 5 min	1.4
8	1-Octanol, Cu(C_5_H_11_COO)_2_, 300 mM	1.7–1.8	EG (40 ml) and hexane (3 ml)	MW heating	220 °C, 5 min	1.4
9	EG, Cu(CH_3_COO)_2_, 100 mM	2.4–2.5	Dodecane (30 ml) and hexane (10 ml)	MW heating	200 °C, 5 min	1.2

a Fiber weight ratio after absorption of raw material solution to dry fiber weight.

To create copper nanoparticles using a hydrophobic raw material solution, 1-octanol solution (Cu(C_2_H_5_COO)_2_·H_2_O 100 mM or Cu(C_5_H_11_COO)_2_ 30, 100, 300 mM) was used as the raw material solution. A small amount of hexane was floated on EG, which was used for pressurization. Other synthesis procedures were the same as those described above.

### Analyses of fabricated fibers

The fiber surface condition was confirmed using SEM (TM-1000; Hitachi High-Tech Corp.) images. Cross-sectional fiber images were also obtained using SEM (S-4800; Hitachi High-Tech Corp). In addition, XRD (SmartLab; Rigaku Corp.) and EDS (QUANTAX400; Bruker Corp.) were applied for structural and elemental analyses. The copper nanoparticle sizes were found using TEM (Tecnai G2; FEI Co). After the fiber sample was ground in a mortar, it was dispersed in ethanol and dropped on the observation grid. An ATR-FTIR spectrum (Spectrum 100; PerkinElmer, Inc.) was used for structural analysis of cellulose in cotton fibers. For washing tests to compare the apparent color, washing was performed for 30 min with water at 40 °C using a commercial washing machine. Dehydration was performed for 5 min. This cycle was repeated eight times during the total 240 min washing time. After the fibers were cut off during washing, the respective appearances of materials were compared. Furthermore, the cotton fiber was immersed in a nitric acid solution after washing. The copper component of the extract solution was evaluated using ICP-optical emission spectrophotometer measurements (ICP-OES, SPS3100 SII; Hitachi High-Tech Science Corp).

### Antiviral evaluations of fabricated fibers

Antiviral evaluation was performed at the BOKEN Quality Evaluation Institute using plaque assay (Japanese Industrial Standard JIS L 1922:2016), which corresponds to ISO 18184:2014 (MOD). As a procedure for experimentation, we first put the test piece (0.4 g) and test virus solution (0.2 ml) in a vial and incubated them at 25 °C for 2 h. Influenza A virus (H3N2, ATCC VR-1679) was used as a test strain. To wash the virus out from the sample, 20 ml of SCDLP medium was added. The viral infectivity value of the washed out solution was measured using plaque assay. Then the antibacterial activity value was calculated using the following formula.Antiviral activity value = log(control sample, infection value immediately after inoculation) − log(test sample, infectious value after 2 h of action)

Antiviral activity value of 2.0 or higher is regarded as effective. A value of 3.0 or higher is regarded as having a sufficient effect.

## Results and discussion

**Fig. 2:**
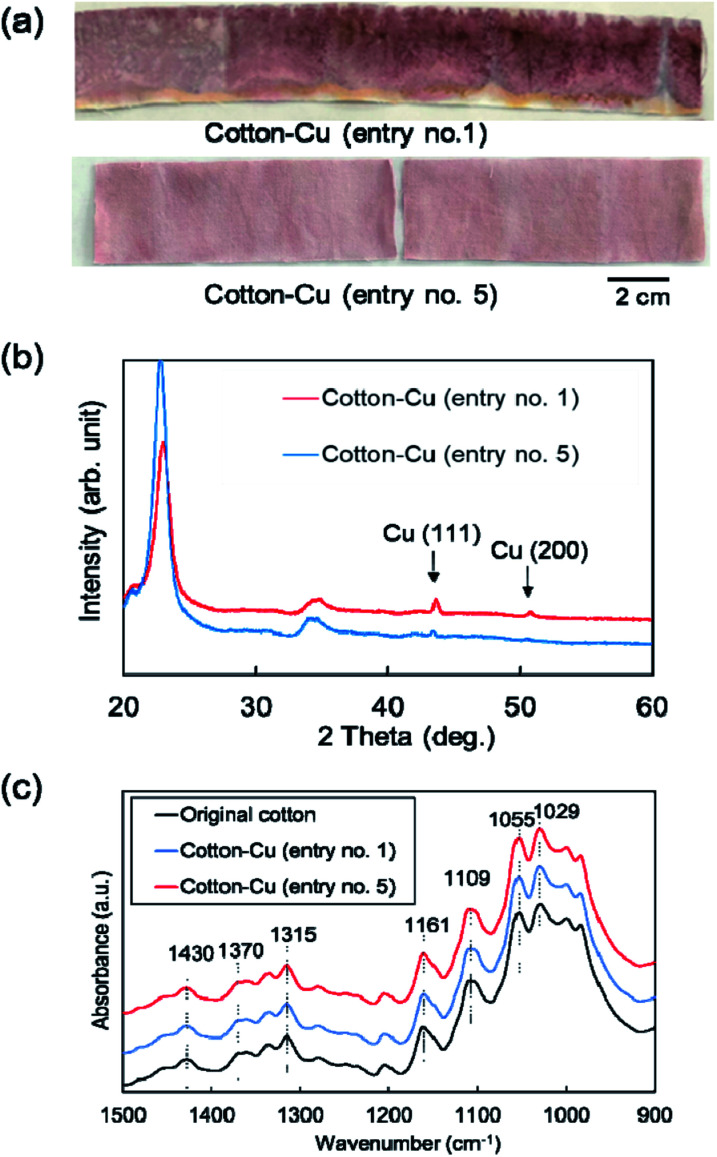
(a) Appearance photograph, (b) XRD patterns, and (c) ATR-FTIR spectra for the fabricated cotton fibers (entry no. 1 and 5).

Fig. 2a portrays the appearance of the fabricated fibers. Both fibers exhibited a distinctive red color because of the surface plasmon resonance of copper nanoparticles. The XRD patterns of these fibers are presented in Fig. 2b. The peaks at 43° and 51° are assigned respectively to the Cu(111) and (200) planes, meaning that these fibers contain copper crystalline particles. These results indicate that copper nanoparticles were fabricated directly into cotton fibers using only polyol or alcohol reduction without a stabilizing agent or a protective agent against aggregation and oxidation of nanoparticles. Fig. 2c depicts the ATR-FTIR spectra of cotton fibers containing copper nanoparticles. In the original cotton fiber and MW fabricated cotton fibers, each absorption peak of 1430 cm^−1^ and 1315 cm^−1^ (C–H wagging), 1370 cm^−1^ (C–H bending), 1109 cm^−1^ (C–O–C asymmetric bridge stretching), and C–O stretching of 1161 cm^−1^, 1055 cm^−1^ and 1029 cm^−1^, which reflect cellulose characteristics, was confirmed.^50,51^ No change was observed in the absorption peak before or after MW heating, suggesting that the chemical structure of cellulose was almost unchanged. In addition, in the ATR-FTIR spectra over various wave numbers shown in Fig. S1,† no additional peak derived from functional groups was observed before and after MW heating.

**Fig. 3:**
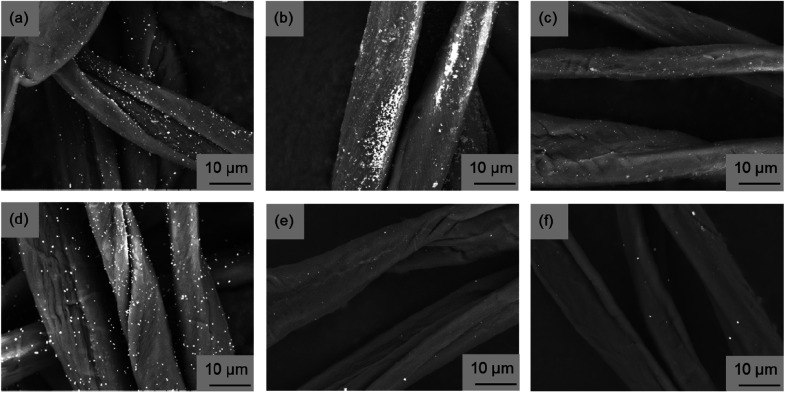
Surface SEM images of the fabricated cotton fibers. (a) MW heating fabrication and (b) electric furnace fabrication using hydrophilic ethylene glycol raw material solution (Cu(CH_3_COO)_2_ 100 mM, entry no. 1, 2). (c) MW heating fabrication using hydrophilic ethylene glycol raw material solution (Cu(CH_3_COO)_2_ 10 mM, entry no. 3). (d) MW heating fabrication using hydrophilic ethylene glycol raw material solution (Cu(NO_3_)_2_·3H_2_O 100 mM, entry no. 4). (e) MW heating fabrication using hydrophobic octanol raw material solution (Cu(C_2_H_5_COO)_2_·H_2_O 100 mM, entry no. 5) or (f) Cu(C_5_H_11_COO)_2_ 100 mM, entry no. 6).

Fig. 3 portrays SEM images obtained for the fabricated fiber surface. Copper nanoparticles were observed as white dots on the fiber surface. Regarding the cotton fibers fabricated using the EG raw material solution, it was confirmed that the MW synthesis tended to have fewer copper nanoparticles on the fiber surface than the reference synthesis using an electric furnace (Fig. 3a and b). Therefore, results confirmed that copper nanoparticles tended to be fabricated inside of the cotton fiber when using this MW method. However, even if the copper raw material concentration was reduced to 1/10 and the absorption amount of the raw material solution was halved, or if the copper raw material was changed from Cu(CH_3_COO)_2_ to Cu(NO_3_)_2_·3H_2_O, the fabrication of copper nanoparticles on the fiber surface was not suppressed by the EG material solution (Fig. 3c and d). The general strategy for imparting functionality to cotton fibers is to create functional substances strongly inside or outside of them by oxidizing or cationizing the hydroxyl groups of cellulose included in cotton fibers.^6^ In our method, it was speculated that the tendency of the hydroxyl groups of the fibers to bond with the hydrophilic raw material solution, which is useful in general functionality-imparting methods, presents a barrier: when the raw material solution was pressurized with the immiscible liquid, presumably the raw material solution remained partially on the fiber surface, leading to copper nanoparticle fabrication on the fiber surface. Therefore, using the opposite combination, *i.e.* a hydrophobic raw material solution, pressure was applied with a hydrophilic liquid. Then MW heating was conducted. In SEM images of fibers fabricated using the two types of hydrophobic raw material solutions shown in Fig. 3e and f, almost no particle fabrication was observed on any fiber surface. Therefore, this idea for suppressing particle fabrication on the fiber surface is probably a general strategy that is independent of the raw material species. Fig. S2† presents a photograph showing the appearance and SEM images of cotton fibers fabricated using Cu(C_5_H_11_COO)_2_ hydrophobic solutions having different copper concentrations. As the copper concentration increased, the red color became darker, indicating increased copper nanoparticle content. From the surface SEM images, copper nanoparticles on the fiber surface were only slightly observed at a copper concentration of 30 mM. In fact, copper nanoparticles on the surface were few even at 300 mM. These results demonstrate that copper nanoparticles can be fabricated inside the fiber over a wide range of copper concentrations.

**Fig. 4:**
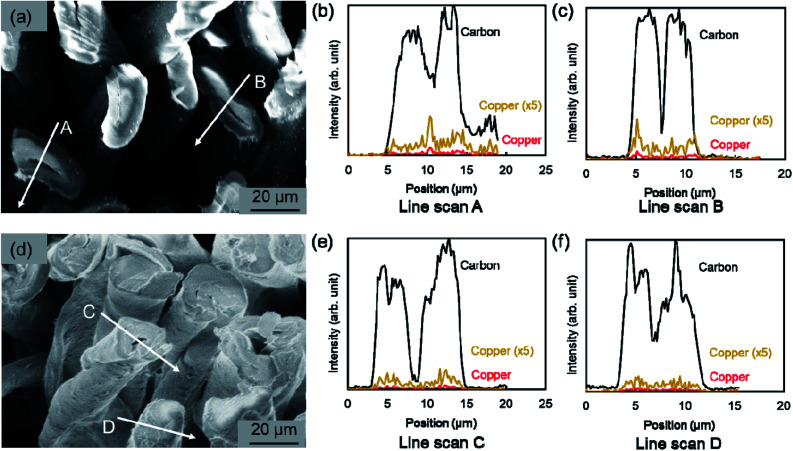
Cross-sectional SEM images and elemental distribution observed using EDS for the fabricated cotton fibers. (a–c) MW heating fabrication using hydrophilic ethylene glycol raw material solution (entry no. 1). (d–f) MW heating fabrication using hydrophobic octanol raw material solution (entry no. 5). The copper component distribution depicts the raw data and the distribution obtained by multiplying the raw data 5 times.

**Fig. 5:**
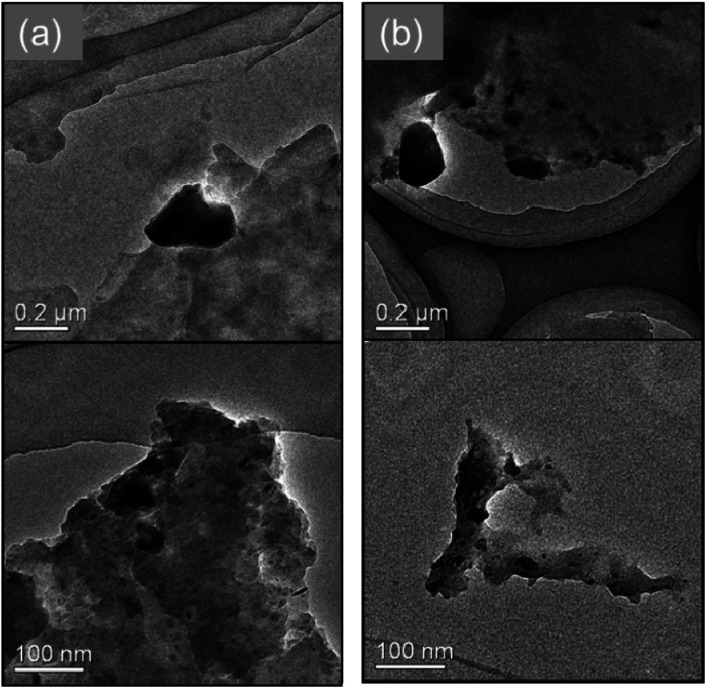
TEM images for the fabricated cotton fibers. (a) MW heating fabrication using hydrophilic ethylene glycol raw material solution (entry no. 1). (b) MW heating fabrication using hydrophobic octanol raw material solution (entry no. 5).

Furthermore, using SEM-EDS, the tendency to suppress copper nanoparticle formation on the surface was clarified. Fig. 4 presents the composition distribution of the fiber cross-section obtained using SEM-EDS. The copper component was distributed inside the carbon component which reflects the cotton fiber. Moreover, when using the hydrophobic raw material solution, the copper component was better distributed inside the cotton fiber, which shows good agreement with results depicted on SEM surface images presented in Fig. 3. The TEM observation results show that copper nanoparticles of 5–30 nm and *ca.* 200 nm were included in both MW-fabricated fibers (Fig. 5). Earlier studies examining silver nanoparticles created inside cotton fibers revealed nanoparticles of about 10 nm, which are of equal size to the microfibrillar substructures.^12,13^ In the MW fabrications described herein, it is presumed that larger copper particles were observed because the particles were present on the fiber surface and on the inner surface of the fiber hollow portion in addition to nanoparticles because of the microfibrillar structure. Regarding the distribution control of the copper component in the fiber cross-section, the elemental profile of Fig. 4 shows that the copper component distribution and strength differed among fibers. The difference occurred because the raw material solution was absorbed and pressurized in the form of a woven fabric. Therefore, the absorption amount of the raw material solution and the pressure amount from the immiscible liquid for each fiber were non-uniform. This fabrication method is applicable to woven fabrics and to yarns or fine fibers.^19^ Controlling the copper component amount and position might be possible to by fabricating each cotton fiber so that the pressure and the absorbed raw material amount can be controlled.

When fabricating copper nanoparticles inside the cotton fiber, a key point is application of heating conditions that are almost identical to the conditions for synthesizing copper nanoparticles using only the raw material solution for fabrication inside of the cotton fiber. When the cotton fiber absorbed the Cu(CH_3_COO)_2_ hydrophilic solution was heated by MW at 200 °C, yellow color that is peculiar to surface plasmon resonance of Cu_2_O nanoparticles was observed (Fig. S3,† entry no. 9). When an EG solution containing Cu(CH_3_COO)_2_ was synthesized using flow-type MW heating in our earlier study, Cu_2_O nanoparticles were synthesized at 180 °C; copper nanoparticles were synthesized at 210 °C.^15^ These conditions resemble those in which copper and Cu_2_O nanoparticles are fabricated inside the cotton fiber. The reaction operation of this *in situ* fabrication method is not limited to MW heating.^19^ When using a method other than MW heating, one must first study the conditions under which copper nanoparticles are synthesized using only the raw material solution and apply those conditions to fabrication inside of the cotton fiber.

**Fig. 6:**
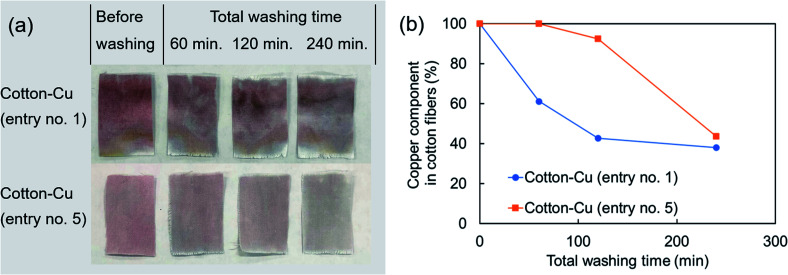
(a) Photograph of the appearance after washing test and (b) time variation of copper component in the fabricated fibers investigated using ICP-OES (entry no. 1, 5).

After a simplified washing test was applied to these fibers, the time variation of the copper component was investigated using ICP-OES (Fig. 6). The appearance after washing revealed a red color, indicating that it contained metallic copper nanoparticles. The decrease of the copper component was slower in the fabricated fiber using the hydrophobic raw material solution than in the hydrophilic raw material solution, indicating good correspondence with the copper distribution of the SEM image surface and elemental mapping in SEM-EDS. The ATR-FTIR spectra indicate that the chemical state of cellulose has changed only slightly. Therefore, the bond between cellulose fibers and copper nanoparticles might be weak. That weakness might be related to reduction in the copper component by this washing test. Early studies have examined nanoparticles with enhanced bonding by chemical treatment of fibers or use of fiber structures to immobilize nanoparticles.^12,52^ It will probably be necessary to combine these methods to increase the durability of the acquired function in our method further. It is noteworthy that copper nanoparticles on the fiber surface remained even after washing tests (Fig. S4†). In this regard, a reducing functional group (aldehyde group) is known to be present at the end of the molecular chain of cellulose and is known to have a weak reducing ability.^53,54^ Copper nanoparticles dotted on the fiber surface were observed, probably because the copper ions remaining on the fiber surface reacted with the reducing functional group of the cellulose and chemically bonded thereafter.

The amounts of copper components leached in the washing test were *ca.* 200 mg kg^−1^ of cotton fibres for 120 min and *ca.* 900 mg kg^−1^ of cotton fibres for 240 min (entry no. 5). To some extent, copper is necessary for human metabolism.^47^ However, in excessive amounts, it has adverse effects. In recent years, many reports have described the safety of nanoparticles. It has been pointed out that water containing copper of 30 mg l^−1^ or more has adverse effects on human health.^46^ The leached amount of copper must be controlled to limit effects on the human body according to the application. Furthermore, nanoparticle safety requires assessment not only of direct effects on the human body, but also assessments including aquatic organisms and the natural environment.^55,56^ Reportedly, copper nanoparticles are more toxic when released as particles than when eluted as copper ions.^57^ Regarding the release mechanism of the copper component of our fabricated fibers, we evaluated neither the ratio of the leaching amount as copper ions nor the elimination amount as copper nanoparticles. However, our method can suppress the adhesion of particles to the fiber surface. Therefore, it has some potential to reduce particle elimination.

Finally, antiviral tests against influenza A virus were conducted for fibers fabricated with included copper nanoparticles (Table 2). Antiviral activity values of the fabricated fibers were found to be higher than the reference value of 3.0, indicating high antiviral properties: results confirmed that the antiviral functionality of the copper nanoparticles was exhibited even in this fabrication method of creating the copper nanoparticles inside of the cotton fiber. The fabricated fibers suppress the formation of copper nanoparticles on the fiber surface. They also have a certain degree of washing durability. Therefore, the fibers are presumed to exhibit antiviral properties while suppressing contact between human skin and copper metal. Moreover, they can be reused while washing repeatedly. These evaluations are left as subjects for future work. The antiviral property of the copper surface by so-called “contact killing” has been reported not only against influenza virus but also against norovirus, monkeypox, vaccinia virus, human immunodeficiency virus (HIV), SARS-CoV, and SARS-CoV-2 virus.^46,58–62^ It is expected that the copper-containing cotton fiber is applicable to various viruses by imparting the proper amount of copper nanoparticles according to the contact killing characteristics of the respective viruses.

**Table 2:** Antiviral activity values for the fabricated fibers

Sample	Antiviral activity value
Cotton–Cu (entry no. 1)	4.3
Cotton–Cu (entry no. 5)	3.6

## Conclusions

Copper nanoparticles were created inside of cotton fibers by pressing an immiscible liquid against a raw material solution and by application of MW heating. To suppress the fabrication of copper nanoparticles on the hydrophilic cotton fiber surface, it was effective to use a hydrophobic raw material solution rather than a hydrophilic raw material solution. The fabricated fiber had antiviral properties against influenza A virus and exhibited inherent properties of copper nanoparticles. This method is a versatile method that is effective for imparting various functional materials inside of natural fibers. Therefore, the method is not limited to application with copper nanoparticles. Future studies will be conducted to evaluate the surface properties of fibers produced using this method and thereby to improve their durability.

## Author contributions

Masato Miyakawa: investigation, methodology and writing – original draft; Chizuru Shigaraki: investigation and visualization; Takashi Nakamura: investigation, resources and writing – review and editing; Masateru Nishioka: conceptualization, supervision and writing – review and editing. All authors approve the publication of the manuscript in the current version.

## Conflicts of interest

There are no conflicts to declare.

## Supplementary Material

RA-011-D1RA04868F-s001
