# Supplementary material for: In situ microwave heating fabrication of copper nanoparticles inside cotton fiber using pressurization in immiscible liquids with raw material solutions
Source: RSC Adv. 2021 Oct 4;11(52):32541–8. doi: 10.1039/d1ra04868f (PMC9042215; doi:10.1039/d1ra04868f)
Supplement: RA-011-D1RA04868F-s001 [file RA-011-D1RA04868F-s001.pdf]

Electronic Supplementary Material (ESI) for RSC Advances.
This journal is © The Royal Society of Chemistry 2021

Supporting information

In-situ microwave heating fabrication of copper nanoparticles inside cotton fiber using pressurization in immiscible liquids with raw material solutions

Masato Miyakawa, Chizuru Shigaraki, Takashi Nakamura and Masateru Nishioka *

National Institute of Advanced Industrial Science and Technology, AIST, 4-2-1, Nigatake, Miyagino-ku, Sendai, 983-8551, Japan

E-mail: m-nishioka@aist.go.jp

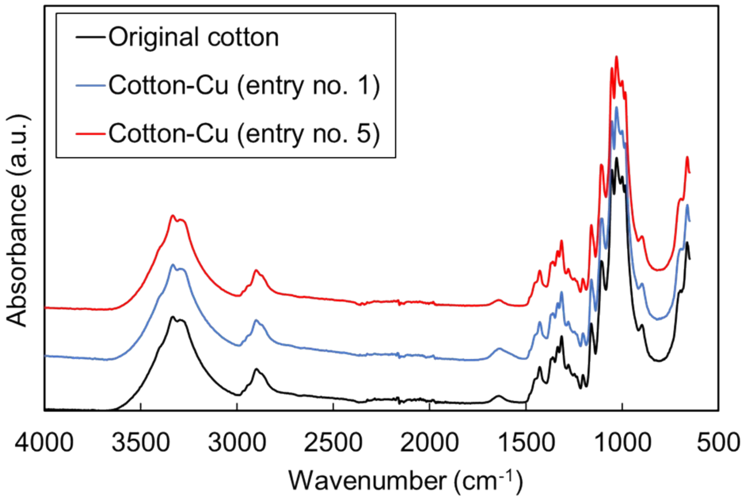

Figure S1. ATR-FTIR spectra of the fabricated cotton fibers.

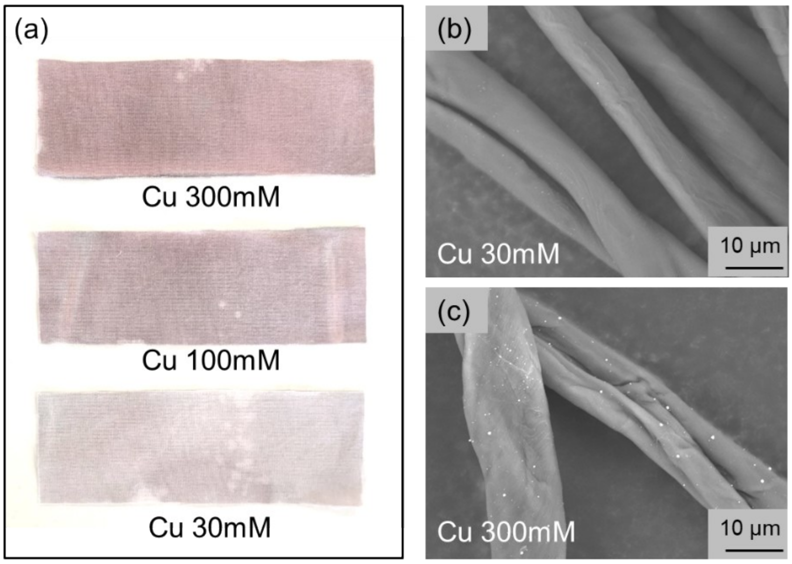

Figure S2. (a) Appearance photograph and (b, c) SEM images of the cotton fibers fabricated using $Cu(C_5H_{11}COO)_2$ hydrophobic solutions with different copper concentrations (entry nos. 6–8).

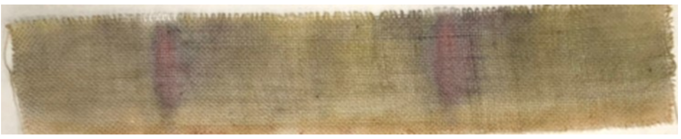

Figure S3. Appearance photograph of the cotton fiber fabricated using $Cu(CH_3COO)_2$ hydrophilic solution. Fabricated temperature by MW was 200 °C (entry no. 9).

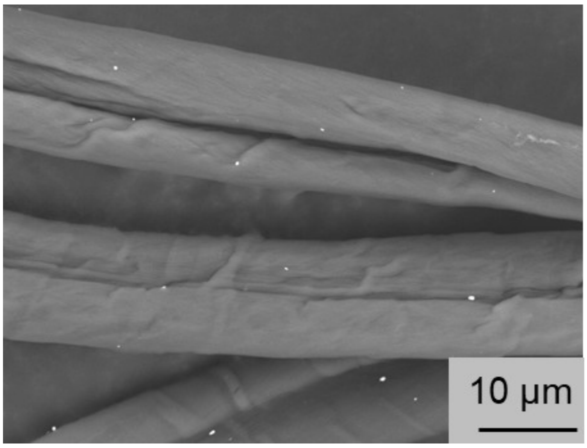

Figure S4. SEM image of the fabricated cotton fiber after 240 min washing test (entry no. 5).
